# Image-Based Three-Dimensional Analysis to Characterize the Texture of Porous Scaffolds

**DOI:** 10.1155/2014/161437

**Published:** 2014-06-05

**Authors:** Diana Massai, Francesco Pennella, Piergiorgio Gentile, Diego Gallo, Gianluca Ciardelli, Cristina Bignardi, Alberto Audenino, Umberto Morbiducci

**Affiliations:** ^1^Department of Mechanical and Aerospace Engineering, Politecnico di Torino, Corso Duca degli Abruzzi 24, 10129 Turin, Italy; ^2^Centre for Biomaterials & Tissue Engineering, Department of Adult Dental Care, School of Clinical Dentistry, University of Sheffield, Sheffield S10 2TA, UK

## Abstract

The aim of the present study is to characterize the microstructure of composite scaffolds for bone tissue regeneration containing different ratios of chitosan/gelatin blend and bioactive glasses. Starting from realistic 3D models of the scaffolds reconstructed from micro-CT images, the level of heterogeneity of scaffold architecture is evaluated performing a lacunarity analysis. The results demonstrate that the presence of the bioactive glass component affects not only macroscopic features such as porosity, but mainly scaffold microarchitecture giving rise to structural heterogeneity, which could have an impact on the local cell-scaffold interaction and scaffold performances. The adopted approach allows to investigate the scale-dependent pore distribution within the scaffold and the related structural heterogeneity features, providing a comprehensive characterization of the scaffold texture.

## 1. Introduction

Tissue engineering scaffolds are designed to provide a biomimetic three-dimensional (3D) architecture that mimics the native extracellular matrix (ECM), in order to guarantee adequate mechanical support and to promote cell colonization, migration, and proliferation. In parallel, such an architecture must assure an adequate oxygen and nutrient diffusion and the removal of metabolic wastes [1, 2].

Both mechanical properties and the above mentioned transport phenomena are strictly dependent on scaffold structure, with porosity, pore size, and specific surface area that play an essential role in cell migration, tissue in-growth, and cell attachment, respectively [3]. Moreover, interconnectivity and distribution of pores strongly affect tissue regeneration [4].

It is widely accepted that highly porous scaffolds with uncontrolled architecture do not recapitulate the desired features of the native ECM/tissue [5], which contrarily assures, with a 3D interconnected and homogeneous pore network, spatially uniform cell distribution, cell survival, proliferation, and migration [6].

In native tissue, structure and function are highly interrelated, therefore an in-depth analysis of the texture of porous scaffolds could allow us to get more insight into the comprehension of the impact that the scaffold architecture has in conditioning (1) not only the cellular environment and cell-cell interactions but also (2) the local cell-structure interactions. It is then clear the relevance of scaffolds microarchitecture when the final aim is to design and build effective functional substitutes [5].

The need to characterize the texture of an object at different scales and to quantitatively assess its spatial patterns is a critical issue for a huge amount of processes in many research fields, from landscape ecology [7] to the analysis of microvascular remodeling [8] and to the study of water movement in relation to soil macroporosity [9], and, in general, for all those porous media that exhibit significant physical heterogeneities, leading to the development of a wide number of metrics. However, most of these metrics suffer from the limitation that different spatial patterns can be depicted for any single value of the respective metric [7]. For example, Mandelbrot recognized that objects with identical fractal dimensions can have greatly different appearances [10], and experience has demonstrated that the classical fractal dimensions are not sufficient to describe uniquely the interstitial geometry of porous media [11]. Indeed, although porous media, and porous scaffolds as well, could be considered as fractal structures, the fractal dimension alone is not sufficient for characterizing scaffolds architecture, since it describes how much space is filled but does not indicate how the space is filled by the object [8].

To overcome this limitation, a new concept, termed lacunarity, was introduced by Mandelbrot [10]. Lacunarity measures the deviation of a geometric object from the translational invariance or homogeneity [12] and can be used to describe the distribution of gap or pore sizes within the studied object [8], characterised by higher lacunarity values if pore sizes are distributed over a greater range. Lacunarity can be adopted to distinguish objects with similar fractal dimensions [8] but can also be used independently to describe spatial patterns [13]. In other words, as translational invariance is a highly scale-dependent property (i.e., objects which are homogeneous at a certain scale could be characterized by heterogeneity at a different scale) [7], lacunarity, which can be considered a scale-dependent measure of heterogeneity, represents an effective tool to study the scale-dependent pore distribution patterns within a scaffold and the related randomness spatial scale.

The aim of the present study is to characterize the microstructure of three bioactive glass/polymer composite scaffolds for bone tissue regeneration in order to get insight into their microarchitecture and the related randomness scale. Starting from realistic 3D models of the scaffolds reconstructed from micro-CT images, the level of heterogeneity of scaffolds architecture is evaluated performing an analysis of lacunarity. Moreover, since the scaffolds under investigation are characterized by different porosity, the relative lacunarity function is adopted for a suitable comparison to exclude the influence of the porosity.

## 2. Materials and Methods

All the single steps of the workflow, from scaffolds preparation to micro-CT image analysis, image segmentation, 3D model reconstruction, evaluation of porosity, and evaluation of pore structure distribution will be detailed in this section.

### 2.1. Scaffold Fabrication

A detailed description of the adopted scaffolds can be found in [14]. Briefly, porous scaffolds were made of blends of chitosan/gelatin (CG), for supporting cell adhesion and proliferation, containing different amounts of bioactive glasses (BG), which are inorganic materials stimulating the biomineralization, and were fabricated by freeze-drying. Foams with three different weight ratios (BG/CG) between the components (S1: 0/100 w/w; S2: 40/60 w/w; S3: 70/30 w/w) were prepared. Details on mechanical properties, biocompatibility, and bioactivity of these scaffolds are described in previous study [14].

### 2.2. Micro-CT-Based 3D Scaffold Geometry Reconstruction

Micro-CT images were used to reconstruct 3D models of the scaffolds. The SkyScan 1072 (Aartselaar, Belgium) micro-CT scanner (248 A current, 40 kV voltage) was used to perform the CT scanning of the manufactured scaffolds. Image slices with isotropic voxels were acquired and a spatial resolution of 8.7 *μ*m was achieved.

Micro-CT image segmentation was performed by applying the open public domain Java image processing software* ImageJ* (http://imagej.nih.gov/ij/index.html). The uncertainty in the reconstruction of scaffold models was minimized adopting several local and global segmentation strategies (exhaustive details can be found at http://rsbweb.nih.gov/ij/). The most performing segmentation strategy was identified as the one giving the maximum value of normalized cross-correlation between the Fourier phases of the original image and the segmented one as proposed elsewhere [15]. The calculation of the normalized cross-correlation, performed within MATLAB (The MathWorks, Inc., Natick, USA) environment, allowed the identification of the Niblack segmentation criterion [16] as the most performing one for scaffolds S1 and S2 and the Sauvola criterion [17] for scaffold S3.

**Figure 1:**
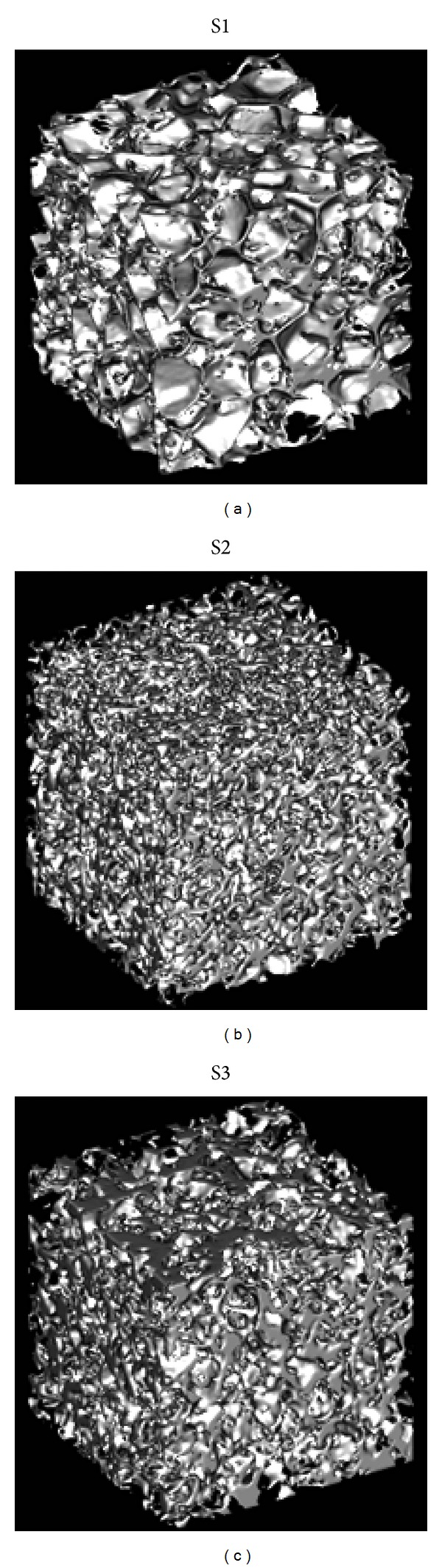
3D model reconstruction from micro-CT images of (a) scaffold S1 (0/100), (b) scaffold S2 (40/60), and (c) scaffold S3 (70/30). Differences in the structure can be observed, which can be ascribed to the different composition of the scaffolds.

The reconstruction of the 3D model of each scaffold was performed from the stack of the properly segmented 2D images. The size of the reconstructed cubic 3D scaffold models from region of interest (ROI) images was equal to 2.2 × 2.2 × 2.2 mm^3^, corresponding to 256^3^ voxels. The 3D volume rendering of the selected ROI of the scaffolds is presented in Figure 1.

### 2.3. Analysis of the Scaffold Architecture: Porosity

As recently mentioned in Pennella et al. [18], properties of a scaffold in terms of mass transport, cell colonization, and mechanical performance can be characterized in statistical terms from its porosity, average pore size, and pore size distribution.

In a widely adopted conceptual model, the internal microstructure of a porous medium (and of a porous scaffold as well) can be characterized by partitioning the pore space into a discrete collection of individual pores, which can be rigorously defined as regions of the void space confined by solid surfaces. In this way, it is possible to define the porosity *n* as follows:

(1) $$\begin{array}{c} n=\frac{V_{V}}{V_{\text{TOT}}}, \end{array}$$

where *V*_*V*_ is the volume of void space and *V*_TOT_ is the total volume [15, 19].

A simple, widely adopted approach based on microscopic surface analysis of the scaffold was applied to evaluate not only porosity, but also the average pore size and superficial pore size distribution [20]. Porosity was evaluated by applying (1) to the reconstructed 3D scaffold models.

### 2.4. Analysis of the Scaffold Architecture: Lacunarity

**Figure 2:**
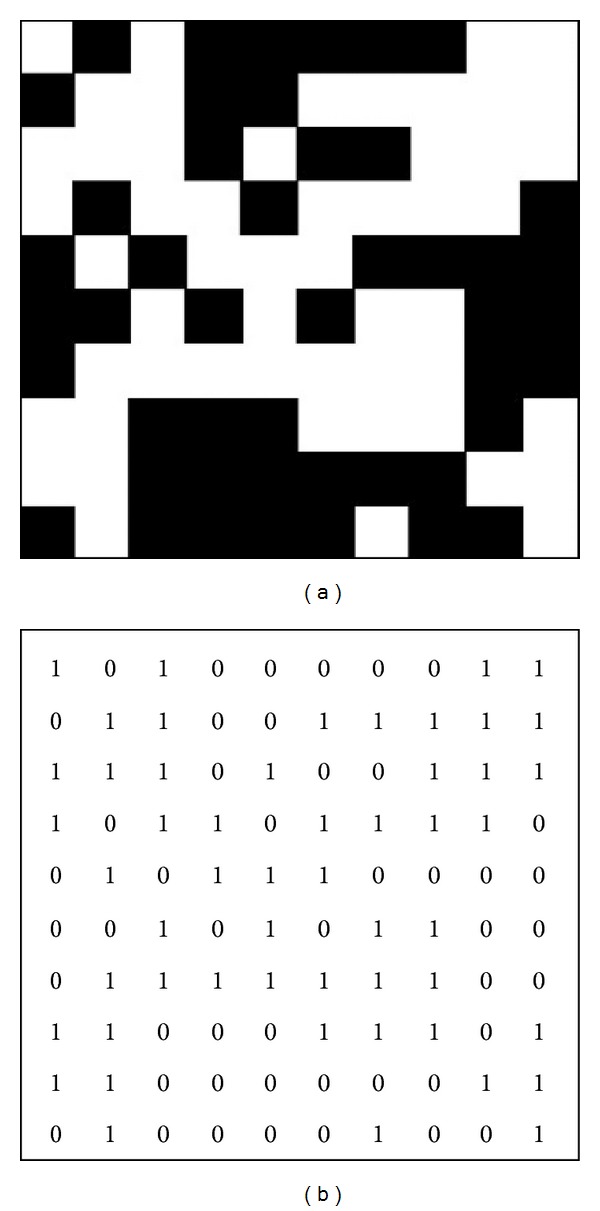
Synthetic 10 × 10 pixel binarized image of a two-dimensional micro-CT slice (a) and its binary representation (b).

A quantitative descriptor of lacunarity was calculated in order to measure the spatial distribution and heterogeneity of scaffold pores. After the segmentation process, each 3D scaffold model, as obtained from the stack of segmented micro-CT images, was converted into a binary map where each grid cell was denoted with zero (black cell, solid space) or one (white cell, pore), obtaining a cube of black and white voxels. An example of a synthetic 10 × 10 pixel binarized image of a two-dimensional micro-CT slice and of its numerical binary representation is presented in Figure 2.

Lacunarity (LAC) was evaluated with the aim to provide an analysis of scaffold images in terms of (1) the overall fraction covered by the attribute of interest; (2) the presence and scale of randomness; (3) the existence of hierarchical structure.

Technically, LAC was evaluated by applying the “gliding box” algorithm [21] as reported by Plotnick et al. [7]. The following strategy was applied: (1) a cubic box of size *r* was superimposed to the 3D scaffold model of size *M*; (2) starting from the upper left-hand corner, the box was moved one unit to the right (with a unit corresponding to the voxel size) and the number of white (pores) voxels contained within the box was counted; (3) the box was shift down one voxel size when the end of a row was reached and the process was repeated until the box was moved over all parts of the cubic 3D scaffold model.

If *N*(*r*) = (*M* − *r* + 1)^2^ is the total number of boxes of size *r* and *n*(*S*, *r*) is the number of boxes of size *r* containing *S* white voxels, then the frequency distribution *N*(*r*) can be converted into a probability distribution as follows:

(2) $$\begin{array}{c} P\left(S,r\right)=\frac{n\left(S,r\right)}{N\left(r\right)}. \end{array}$$

LAC of the 3D scaffold model, for box size *r*, can now be defined as

(3) $$\begin{array}{c} \text{LAC}=\frac{\mu_{2}\left(r\right)}{{\left(\mu_{1}\left(r\right)\right)}^{2}}=\frac{{\overset{-}{S}}^{2}\left(r\right)+\sigma_{s}^{2}\left(r\right)}{{\overset{-}{S}}^{2}\left(r\right)}=1+\frac{\sigma_{s}^{2}\left(r\right)}{{\overset{-}{S}}^{2}\left(r\right)}, \end{array}$$

where *μ*_1_ and *μ*_2_ are the first and second moments of the distribution *P*(*S*, *r*), respectively; and $\overset{-}{S}\left(r\right)$ and *σ*_*s*_^2^(*r*) are the mean and the variance of the number of white (pore) voxels per box of size *r*, respectively.

Equation (3) shows that LAC is a function of *r*. As the size of the gliding box increases, the content of the box also increases and the probability that box contents will greatly differ from the average decreases. This is like to say that also the variance of *S*(*r*) decreases (and *μ*_2_ in (3) as well) with the consequence that the same scaffold model will show lower LAC values as *r* increases. Moreover, there is a clear dependence on LAC from the void fraction of the scaffold model (representing pores). As the mean number of voids goes to zero, the ratio $\left(\sigma_{s}^{2}\left(r\right)/{\overset{-}{S}}^{2}\left(r\right)\right)$ increases in (3), with the consequence that scaffolds with sparse pore distribution will have higher LAC than scaffolds with more dense pore maps, for the same *r*.

LAC is also sensitive to the pore size and distribution within the scaffold: for a given void fraction in the scaffold model, fewer but larger pores give rise to higher LAC values. In contrast, the LAC  value of a totally regular scaffold model is equal to one, independent of the value of *r* (the variance *σ*_*s*_^2^(*r*) is zero at any location, because the number of white voxels within the gliding box is constant).

The above mentioned considerations clearly confirm the observation by Plotnick et al. [7] that an evaluation of lacunarity based on a single gliding box size *r* is meaningless, when LAC is used for comparison of different scaffold models. On the contrary, the possibility to extract a whole host of information is given when LAC is calculated over a wide range of gliding box sizes. This can be done by analyzing the shape of LAC versus the gliding box size *r* curves [7].

The scaffolds under investigation in this study are characterized by different porosity (as will be shown in Section 3), making comparison difficult. However, since the overall shape of the LAC curves depends on the degree of clustering or clumping and is independent of the value of the fraction [7], which in this case is porosity *n* of (1), the relative lacunarity function (RLF) on a logarithmic scale was adopted to minimize the influence of different porosity on scaffolds architecture heterogeneity. According to Luo and Lin [9] RLF was calculated as follows:

(4) $$\begin{array}{c} \text{RLF}=-\frac{\ln\left(\text{LAC}\right)}{\ln\left(n\right)}. \end{array}$$

Using (4), the shape of the RLF versus *r* curve and its corresponding spatial pattern could be better evaluated.

## 3. Results

The porosity values calculated from the reconstructed 3D models of the scaffolds are summarized in Table 1. As expected, porosity *n* for scaffold S1, composed of CG alone without BG component, is greater than that for scaffolds S2 and S3. Interestingly, the porosity of scaffolds containing BG at different percentages (S2 and S3) is almost the same. This result is related to the deposition of the BG particles on the pore walls, which has the consequence of a reduction of the available void area [14].

For each scaffold model, LAC and RLF were calculated for gliding box size *r* ranging from 1 to 64.

**Figure 3:**
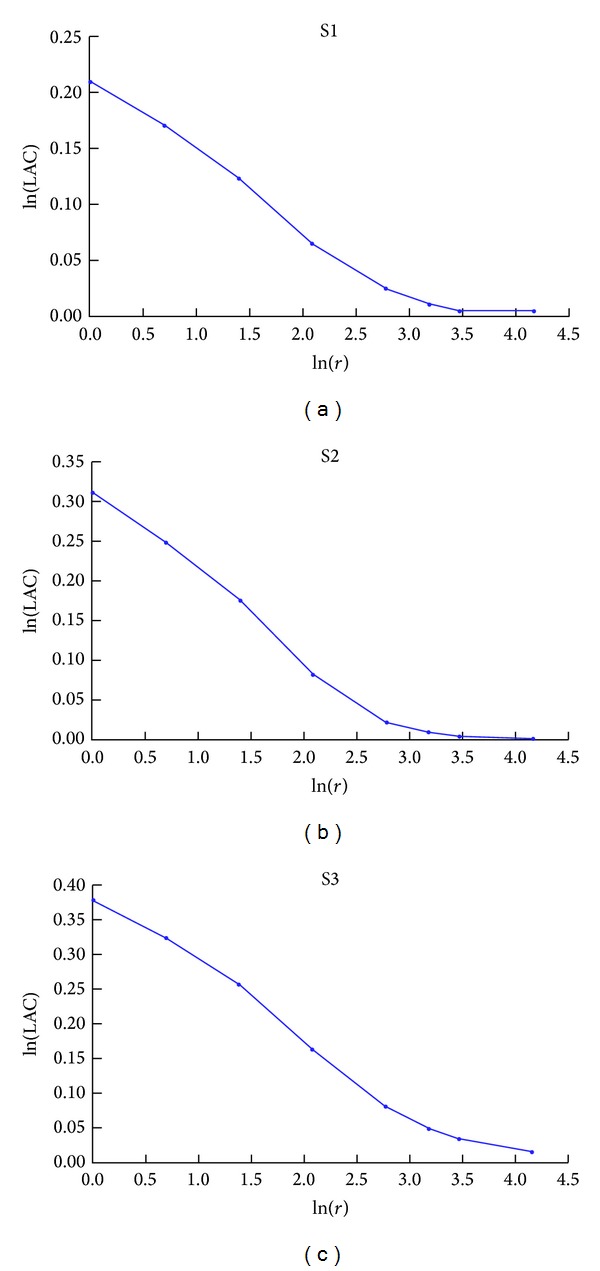
Log-log plot of LAC versus gliding box size *r* calculated over the 3D model of scaffolds S1 (a), S2 (b), and S3 (c).

Figure 3 shows the log-log plots of the scaffold LAC values versus *r* for scaffolds S1, S2, and S3, respectively. It is worth noting that, in general, (1) the maximum LAC value is always found when *r* is equal to 1 (ln⁡(*r*) = 0), because in this case LAC is simply a function of the void/solid fraction, that is, porosity *n*, and does not give information about pore distribution; (2) LAC is always equal to 1 (ln⁡(LAC) = 0) for *r* is equal to the maximum sample size, because in this case the variance *σ*_*s*_^2^(*r*) in (3) is always zero; (3) away from the endpoints, in general, the shape of LAC curves differs also when the same fraction of the 3D scaffold model is occupied by voids, that is, also when scaffolds have the same porosity but different pore distribution, shape, dimension, and so forth.

From our findings, it can be observed that LAC curves are not linear (Figure 3), thus clearly indicating that scaffolds are not characterized by a fractal geometry. In fact, when a porous media is characterized by self-similarity, LAC log-log plot should be linear [7, 21]. As reported by Plotnick et al. [7], if a map has a random structure at some scale, lacunarity depends on the size *r* of the gliding box relative to the characteristic scale of randomness. In particular, if *r* is greater than the random scale, the variance of the void space (pores) within the gliding boxes will approach zero and LAC will be close to 1, while if *r* is smaller than the scale of randomness, LAC will be higher than 1 pointing out heterogeneity. Considering Figures 3(a) and 3(b), it is possible to observe that for scaffolds S1 and S2 the LAC curves begin to approach a value close to 1 when ln⁡(*r*) ≈ 2 (i.e., *r* ≈ 8 voxels, corresponding to 64 *μ*m), as highlighted by the change in the slope of the curve. This means that at scales lower than ln⁡(*r*) ≈ 2, scaffolds S1 and S2 are characterized by an heterogeneous structure with random patterns. Moreover, both for S1 and S2, LAC approach values close to one 1 for ln⁡(*r*) > 4. Scaffold S3 exhibits a similar trend as for S1 and S2, but a slower LAC decrease with *r* (Figure 3(c)), thus indicating a slightly wider scale of randomness for S3 structure.

The results in Figure 3 show that the gliding box size *r* has approached the representative elementary volume (i.e., *r* ≈ 64) of the reconstructed scaffolds [9].

**Figure 4:**
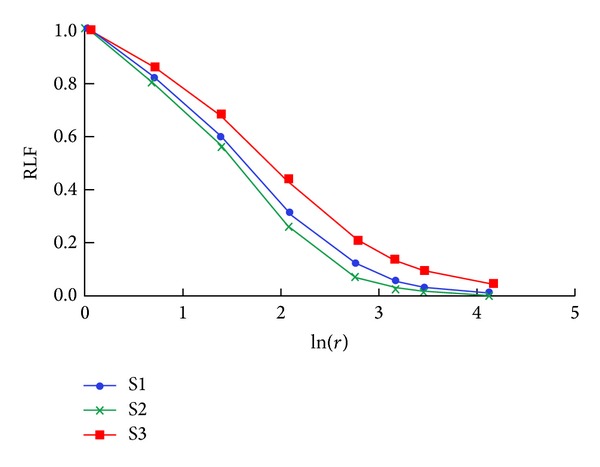
Log-log plot of RLF versus gliding box size *r* calculated over the 3D models of scaffolds S1, S2, and S3.

As scaffolds are characterized by different porosity, in order to compare their structural heterogeneity, the RLF was calculated, and information about their randomness scale was obtained by analyzing the shapes of RLF versus ln⁡(*r*) curves. The RLF curves of scaffolds S1, S2, and S3 are depicted in Figure 4.

**Figure 5:**
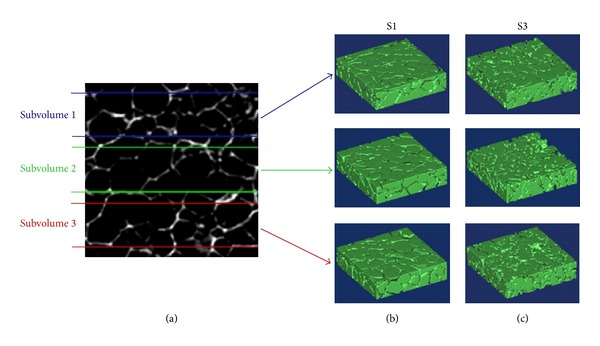
3D reconstruction of three (parallel) subvolumes, obtained from different regions (a) of scaffold models S1 (b) and S3 (c).

Scaffolds S1 and S2 are characterized by the same trend, with the RLF curve of S2 being always lower than S1. This could be related to the increased wall thickness of pores in S2, a consequence of the deposition of BG particles. Concerning S3, it is characterized by RLF values higher than S1 and S2, independent of *r*. This means that S3 exhibits a more marked heterogeneity than S1 and S2, which could be ascribed to its higher content of BG particles that causes micropore occlusion. To further investigate the reason for this behaviour, three (parallel) subvolumes (thickness = 520 *μ*m), obtained from three different regions of the original reconstructed scaffold model, were considered both for S1 and S3, and on them the RLF was calculated (Figure 5).

**Figure 6:**
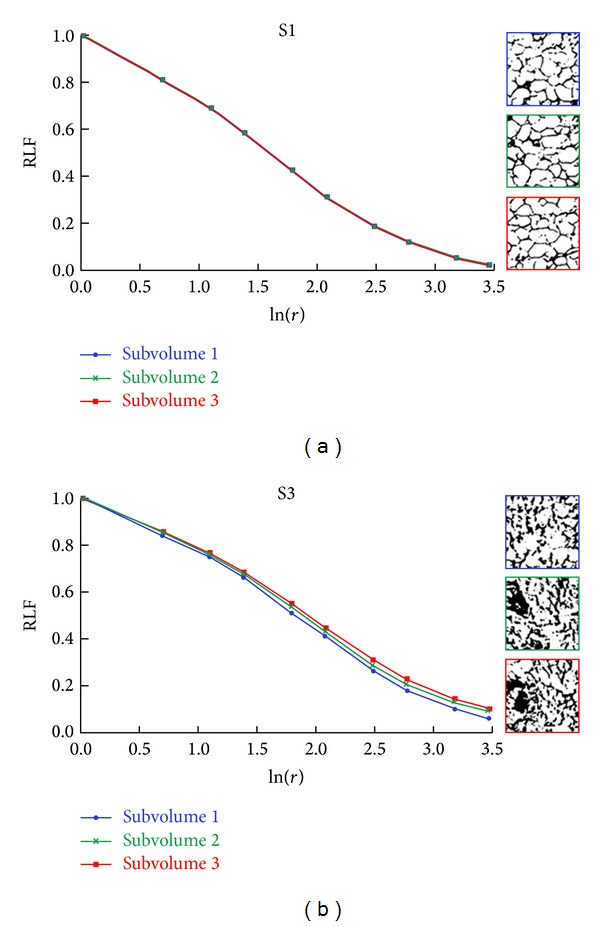
Log-log plot of RLF versus gliding box size *r* of three different subvolumes of scaffolds S1 (a) and S3 (b). The binarized images of the pore network, related to the three subvolumes under investigation, are also shown.

The log-log plot of RLF versus *r* values for the three subvolumes of S1 and S3 is displayed in Figure 6. Notably, no differences can be appreciated in the curves of the three subvolumes belonging to S1 (Figure 6(a)). On the contrary, RLF curves of the three subvolumes belonging to S3 show remarkable differences for ln⁡(*r*) > 0.5 (Figure 6(b)), thus confirming dissimilar spatial distribution of pores within different regions of the scaffold. More in detail, for S3 the RLF curve of subvolume 1 shows lower values than curves of subvolumes 2 and 3, indicating the presence of distributed pores and/or less pore occlusion, as also confirmed by the visual inspection of the binarized images of the pore network related to the investigated subvolumes, depicted in Figure 6(b).

## 4. Discussion

In a large part of tissue engineering approaches, the microarchitecture of porous scaffolds plays a key role in effectively guiding cell growth and tissue regeneration. The architecture is in fact among the main contributors in determining the performance of the scaffold itself in terms of both adequate mechanical support and transport of cells and compounds [1, 2].

It is then clear that in scaffold architecture the level of heterogeneity rather than self-similarity could have marked side effects on the quality of the engineered tissue, eventually giving also rise to scale effects.

In parallel to the development of tools for the macroscopic characterization of scaffold features (e.g., Young modulus, porosity, permeability, etc.) [18, 22], all the reasons mentioned above have lead, in recent years, to an increasing interest in (1) methods for characterizing scaffold architecture at different scales [5, 23] and in (2) quantitative descriptors of spatial emerging patterns in scaffold structure [24].

Inspired by methods applied in other disciplines [7–9], in this study a method based on lacunarity analysis was adopted for a quantitative description of the texture of three glass/polymer composite porous scaffolds for bone tissue engineering and for the identification of their randomness scale.

The effectiveness of the approach allowed to assess the spatial distribution of pores over the 3D reconstructed scaffold models and to catch heterogeneity features in the structures of the three investigated scaffolds which, due to their composition and fabrication method, lack self-similarity.

Interestingly, the findings demonstrate that the presence of the BG component affects not only porosity (S1, the scaffold composed of chitosan/gelatin alone is characterized by higher porosity than S2 and S3) but also mainly heterogeneity. In fact, the scaffold with the highest BG content, S3, presents higher spatial heterogeneity than S1 and S2, as confirmed by the RLF analysis (Figure 4). This result is independent of porosity, being S2 and S3 characterized by almost the same porosity, which is markedly lower than S1 (Table 1). Moreover, the analysis performed on subvolumes of scaffolds S1 and S3 highlights (1) different levels of heterogeneity in different regions of S3 (Figure 6(b)), against the same levels characterizing different regions of S1 (Figure 6(a)) and (2) a scale of randomness for S3 which is slightly wider than that for S1 (Figure 6).

Previous findings on the same scaffolds showed that one consequence of the increased presence of BG in the composition of the scaffold was a structure more resistant to compression [14]. Also in this case, it is expected that heterogeneity in the microarchitecture could play a scale effect, thus contributing to the increase of the anisotropic mechanical behaviour of the scaffold.

The approach applied in this study could suffer from limitations. A possible limitation of the adopted method could be in the fact that, being the lacunarity analysis based on images, results could be markedly influenced by the adopted image resolution [11]. However, in this specific study, the high resolution (8.7 *μ*m) of the micro-CT images is adequate with respect to the mean pore size (greater than 130 *μ*m [14]; that is, mean pore size is 15 times higher than micro-CT image resolution) of the scaffolds under investigation. A further possible limitation can be identified in the uncertainty in the reconstruction of the 3D models, which could affect the analysis of the texture. Also in this case, we put effort in selecting, among several possible segmentation strategies (as explained in Section 2), the most appropriate one, thus minimizing the impact that this source of uncertainty could have on texture analysis.

## 5. Conclusions

In the present paper, the textures of three glass/polymer composite porous scaffolds for bone tissue engineering were characterized by adopting an image-based method based on lacunarity analysis. Our findings suggest that the texture of porous scaffolds could play a crucial role in determining the properties of the structure not only at the macroscale, but also at lower scales, where the focal relationships between cells and structure take place. The approach herein applied to engineered scaffolds could be translated to the microstructure of the native ECM of different tissues [25] in order to (1) investigate its local effects on the relationship between cell and ECM [26] and (2) design and fabricate biomimetic porous scaffolds that recapitulate the ECM architectural features of the tissue of interest [5].

In the future, 3D metrics for the analysis of spatiotemporal data as developed in ecology will be applied to 3D models of scaffolds as reconstructed from, for example, coherent anti-Stokes Raman scattering microscopy images [27], along the cell culture. In this way, the evolution of the cultured construct will be evaluated as the evolution of an “ecosystem,” considering the different actors of the involved complex bioprocesses. This approach will allow to identify relationships of relevance between the level of complexity at which the system is considered and the granularity of its description, that is, the so called “contextual emergence” [28]. The proposed ecosystem evolution-like approach, applied to study the evolution of the cell-scaffold system, could provide a robust procedure which, being able to translate between descriptive levels, can be used to build up consistent level-specific criteria for reproducibility.

## Figures and Tables

**Table 1:** Porosity (*n*) values calculated over the 3D models of scaffolds S1, S2, and S3.

Scaffold (BG/CG)	S1 (0/100)	S2 (40/60)	S3 (70/30)
*n* (%)	81	70	68
